# *In silico* Prediction of New Drug Candidates Against the Multidrug-Resistant and Potentially Zoonotic Fish Pathogen Serotype III *Streptococcus agalactiae*

**DOI:** 10.3389/fgene.2020.01024

**Published:** 2020-08-28

**Authors:** Leonardo Mantovani Favero, Roberta Torres Chideroli, Natália Amoroso Ferrari, Vasco Ariston De Carvalho Azevedo, Sandeep Tiwari, Nelson Mauricio Lopera-Barrero, Ulisses de Pádua Pereira

**Affiliations:** ^1^Laboratory of Fish Bacteriology, Department of Preventive Veterinary Medicine, State University of Londrina, Londrina, Brazil; ^2^Institute of Biological Sciences, Department of Genetic, Ecology, and Evolution, Federal University of Minas Gerais, Belo Horizonte, Brazil; ^3^Department of Animal Production, State University of Londrina, Londrina, Brazil

**Keywords:** bioinformatics, core genome, drug discovery, fish disease, molecular docking, streptococcosis, subtractive genomics

## Abstract

*Streptococcus agalactiae* is an invasive multi-host pathogen that causes invasive diseases mainly in newborns, elderly, and individuals with underlying health complications. In fish, *S. agalactiae* causes streptococcosis, which is characterized by septicemia and neurological signs, and leads to great economic losses to the fish farming industry worldwide. These bacteria can be classified into different serotypes based on capsular antigens, and into different sequence types (ST) based on multilocus sequence typing (MLST). In 2015, serotype III ST283 was identified to be associated with a foodborne invasive disease in non-pregnant immunocompetent humans in Singapore, and the infection was related to raw fish consumption. In addition, a serotype III strain isolated from tilapia in Brazil has been reported to be resistant to five antibiotic classes. This specific serotype can serve as a reservoir of resistance genes and pose a serious threat to public health. Thus, new approaches for the control and treatment of *S. agalactiae* infections are needed. In the present study, 24 *S. agalactiae* serotype III complete genomes, isolated from human and fish hosts, were compared. The core genome was identified, and, using bioinformatics tools and subtractive criteria, five proteins were identified as potential drug targets. Furthermore, 5,008 drug-like natural compounds were virtually screened against the identified targets. The ligands with the best binding properties are suggested for further *in vitro* and *in vivo* analysis.

## Introduction

*Streptococcus agalactiae* is a multi-host, invasive, Gram-positive pathogen, identified in several species of terrestrial and aquatic mammals, reptiles, amphibians, and fish (Delannoy et al., 2013). It is a commensal organism of the human gastrointestinal and lower genital tracts, with the potential to cause diseases mainly in newborns, elderly, and individuals with underlying medical conditions (Armistead et al., 2019; Raabe and Shane, 2019). Outbreaks of *S. agalactiae* infection, characterized by septicemia, exophthalmia, and meningoencephalitis, has been reported among both farmed and wild fish species, which leads to high mortality of fishes and serious economic losses (Olivares-Fuster et al., 2008; Mian et al., 2009; Soto et al., 2015).

*Streptococcus agalactiae* strains can be classified into ten serotypes based on their capsular antigens (Slotved et al., 2007). Based on multilocus sequence typing (MLST), the strains are classified into sequence types (ST), which are further grouped into clonal complexes (CC) (Barony et al., 2017). In 2015, an *S. agalactiae* human outbreak in Singapore raised concerns about a specific *S. agalactiae* genotype, serotype III ST283. Although *S. agalactiae* isolated from human and cow hosts had been proven to infect fish (Pereira et al., 2010; Chen et al., 2015), and this ST had already been identified in human (Ip et al., 2006; Salloum et al., 2010) and tilapia (Delannoy et al., 2013) infections, this was the first ever case of human infection linked to consumption of raw farmed fish (Tan et al., 2016). Unlike the general nosocomial *S. agalactiae* infection in humans, this outbreak affected younger, non-pregnant, and immunocompetent individuals also (Kalimuddin et al., 2017). Even though this particular ST emerged as a regional threat, it has already spread and has caused outbreaks among farmed fish in Brazil owing to aquaculture and global food trades (Barkham et al., 2019; Leal et al., 2019), supporting the initial concerns to worldwide public health. In addition, the first Brazilian serotype III isolated from diseased tilapia was reported to be multi-drug resistant (i.e., resistant to ampicillin, norfloxacin, aminoglycosides, fluoroquinolone, sulfamethoxazole, and tetracycline) (Chideroli et al., 2017).

Antimicrobial resistance of pathogens is a critical concern to human and animal health. Furthermore, aquaculture systems and their products have been reported to be hotspots for horizontal transfer of gene, particularly antimicrobial resistance genes, from a donor bacteria (which harbor resistance gene) to the environment’s and/or to consumers and handler’s microbiota (Watts et al., 2017). Therefore, it is important to develop novel strategies for the prevention and treatment of infections by multidrug-resistant pathogens. Integration of bioinformatics tools with metabolomics, proteomics, and comparative genomics is of great value for the identification of drug targets in a pathogen (Mondal et al., 2015); moreover, this strategy can reduce the number of *in vitro* trials, rendering the drug discovery process more economical and less laborious (Timo et al., 2019). Promising results have been obtained by integrating *in silico* and *in vivo* techniques for inhibiting multidrug-resistant pathogens, including *Mycobacterium tuberculosis*, *Staphylococcus aureus*, *Escherichia coli*, and *Vibrio cholerae* (Zang et al., 2016; Sandhaus et al., 2018; Tiwari et al., 2019).

*Streptococcus agalactiae* serotype III serves as a reservoir of antibiotic resistance genes and poses a great threat to public health; hence, newer strategies are required for the control and treatment of *S. agalactiae* infections. Thus, through comparative genomics and bioinformatics approaches, this study screened all the available genomes of *S. agalactiae* isolated from human and fish hosts in GenBank of National Center for Biotechnology Information (NCBI) database for conserved proteins that are not homologous to the host’s proteome and can serve as potential drug targets, and virtually screened for drug-like natural compounds that could bind to the identified drug targets.

## Materials and Methods

### Genomes

From the 114 *S. agalactiae* complete genomes available at the NCBI database, 24 were selected based on the following two inclusion criteria: serotype III strains; and isolates from human (*n* = 19) or fish hosts (*n* = 5). Genomes with lack of information about host source at NCBI Biosample page were not included. Both FASTA and GenBank files were downloaded. For comparative genomic analysis, the complete genome of a non-pathogenic *Streptococcus thermophilus* strain isolated from dairy products was downloaded. The genome information available at NCBI database is summarized in Supplementary Table 1.

### Genomic Analysis

A phylogenetic analysis was performed to select representative genomes for better visualization of the concentric alignment, further described. The genomes of the 24 *S. agalactiae* strains and the *S. thermophilus* strain were submitted to Gegenees v2.2 using the following settings: fragment-size, 500 bp; and step-size, 500 bp. This software performs genomic analysis through whole-genome fragmentation followed by an all-against-all BLAST comparison (Ågren et al., 2012). The output is a heatmap showing the similarity between the genomes. The heatmap was exported as a distance matrix in a nexus file, which served as the input for construction of a phylogenetic tree using SplitsTree4 software (Huson and Bryant, 2006). The neighbor-joining method was employed for phylogenetic tree construction according to Ågren et al. (2012), and *S. thermophilus* served as the out-group.

The Brazilian multidrug-resistant fish isolate strain S73, described by Chideroli et al. (2017), was selected to predict genomic islands (GIs) using the software GIPSy, which predicts pathogenicity, metabolic, resistance, and symbiotic islands (Soares et al., 2016). Next, to visualize the presence of selected drug targets in the predicted GIs, a concentric ring alignment was generated using BRIG software (Alikhan et al., 2011). The following data were used for this alignment: genome of S73 strain as the reference genome (center ring), 32 predicted GIs, 48 essential non-host homologous proteins as drug targets, and a representative genome from each cluster of the phylogenetic tree. When more than one ST or hosts were present within a cluster, a representative from this ST or host was also included. The clusters in the phylogenetic tree and the rings in the alignment were identified with same colors.

For identification of conserved proteins among the genomes, the 24 *S. agalactiae* serotype III FASTA files were submitted to the Orthofinder software under its default parameters to identify the core genome. Through BLAST searches and Markov Cluster Algorithm (MCA), this software infers homologous regions and calculates the orthogroups (Emms and Kelly, 2015). Next, core, shared, and singleton genes were identified using in-house scripts. The first genes were present in all genomes, the following genes were present in few genomes, and the later genes were present in only one genome. The core genes were then subjected to subtractive genomics analysis to identify the most suitable proteins for drug binding analysis.

### Subtractive Analysis of Core Proteins

To eliminate targets with any similarity to the hosts’ proteome, the core proteins were subjected to BLASTp searches against human (*Homo sapiens*, taxid: 9606) and tilapia (*Oreochromis* sp., taxid: 8139) proteomes using the NCBI database. Sequences with any similarity to host proteins were excluded. Using Orthofinder software, proteins that were non-homologous to the human and *Oreochromis* sp. proteome were selected for the subsequent steps.

The software SurfG+ was employed to predict subcellular localization of the proteins based on the identification of peptide signals, retention signals, transmembrane helices, and protein secretion pathways (Barinov et al., 2009). SurfG+ is not downloadable and can be found as a part of the Mature Epitope Density Server at https://med.compbio.sdu.dk/. Only cytoplasmatic proteins were selected for drug discovery owing to their importance in the maintenance of cell viability (Vilela Rodrigues et al., 2019).

Three-dimensional (3D) model structures of cytoplasmatic proteins were predicted by Protein Data Bank (PDB) homology modeling utilizing the MHOLline software^1^. This software combines HMMTOP, BLAST, BATS, MODELLER, and PROCHECK programs to generate 3D protein models with structural and functional information (Capriles et al., 2010). Only models with identity ≥50% and *e*-value ≤0.3 (good to very high-quality sequences, according to MHOLline) were selected for further analysis.

The amino acid sequences of the proteins were subjected to BLASTp search against the Database of Essential Genes^2^ (DEG). This online platform comprises information on essential genes from bacteria, archaea, and eukaryotes, essential non-coding RNAs, promoters, regulatory sequences, and replication origins (Luo et al., 2014). Proteins with bit-score ≥100 and *e*-value ≤10^–4^ were selected for further analysis (Mondal et al., 2015).

Following the subtractive strategy, only proteins in or at the edge of predicted GIs were selected for subsequent analysis. As a final selection criterion, the druggability of the proteins was assessed using the DoGSiteScorer algorithm, available at the ProteinPlus web server^3^. This algorithm identifies potential binding sites (referred to as pockets) in the protein 3D models, provides the amino acid composition of these pockets, and infers a druggability score for each, ranging from zero to one (Volkamer et al., 2012; Fährrolfes et al., 2017). Only proteins with pockets having druggability score ≥0.8 were retained, and from these, only the pocket with the highest score was selected for drug binding analysis.

Two databases were employed to obtain more information about the proteins selected as drug targets. The protein annotation database UniProt (The Uniprot Consortion, 2019) was used to access information regarding the functions of the selected proteins and the pathways in which they were involved; and the DrugBank database (Wishart et al., 2018) was used to determine whether the selected proteins have already been tested as drug targets.

### Drug Binding Analysis

A library of 5,008 natural drug-like molecules was downloaded from the ZINC database in SDF format (Sterling and Irwin, 2015). These ligands were then converted into the PDBQT format required for docking using OpenBabel (O’Boyle et al., 2011) and the python script “prepare_ligand4.py,” described in AutoDockTools MGL^4^ user guide web pages. The proteins selected as drug targets were submitted to AutoDockTools MGL (Morris et al., 2009) for visualization of the previously identified druggable pocket; a grid box containing all the amino acid residues of the pocket was created, and the protein model was converted into the PDBQT format.

The ligand library was screened against each target using AutoDock Vina, and the best 10 ligands were identified based to their binding affinity using the python script “vina_screen_get_top.py” (Trott and Olson, 2009). Next, the Chimera program was applied to visualize the different interactions between the identified ligand and the active site of its target protein (Pettersen et al., 2004). The ligand forming the most number of hydrogen bonds with its target and having the lowest energy binding affinity (kcal mol^–1^) was defined as the best drug candidate (Thomsen and Christensen, 2006).

## Results and Discussion

### Genomic Analysis

**FIGURE 1:**
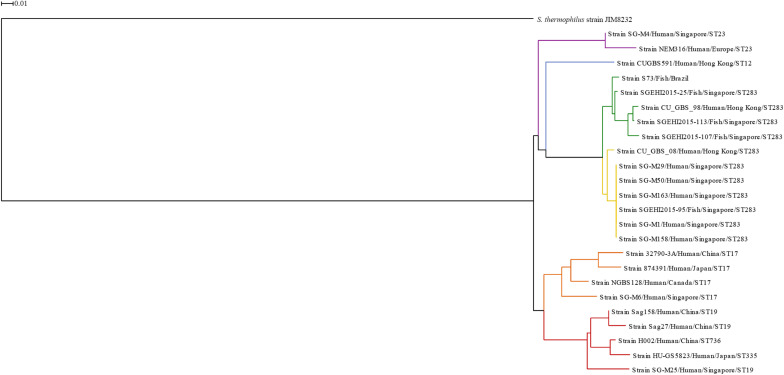
Phylogenetic tree based on whole genomes of *Streptococcus agalactiae* serotype III isolates from human and fish hosts constructed using neighbor-joining method. The scale bar represents a difference of 1% in average BLASTN score similarity. Different colors indicate different clusters. Purple: ST23 cluster; blue: ST12 cluster; green: ST283 subcluster; yellow: ST283 subcluster; orange: ST17 cluster; red: CC19 cluster.

A phylogenetic tree (Figure 1) was constructed based on the heatmap of whole-genome comparison (Supplementary Figure 1) between the 24 *S. agalactiae* serotype III genomes and the *S. thermophilus* genome. It is notable that the genomes of serotype III isolates were found to be highly conserved, and the lowest similarity observed was 82%. In the phylogenetic tree, isolates from the same ST were grouped together. The only two exceptions were strains HU-GS5823 (ST335) and H002 (ST736); though these STs were grouped in the ST19 cluster, they belong to the clonal cluster 19 (Usein et al., 2014; Chen et al., 2015; Emaneini et al., 2016).

The ST283 cluster was divided into two subclusters, one included mainly fish isolates (green subcluster) and the other included human isolates (yellow subcluster); however, in both the subclusters, one isolate from the other species was present. This reinforces the ability and adaptation of this ST to infect both species and cause zoonotic infections, like the Singapore outbreak in 2015 (Tan et al., 2016; Kalimuddin et al., 2017). Furthermore, a phylogenetic tree constructed based on ST283 isolated from human and fish hosts in Southeast Asian countries showed that human and fish isolates were grouped together, with zero single nucleotide polymorphism (SNP) difference in locations where samples were collected together, which was not observed for samples collected from different countries (Barkham et al., 2019). In the phylogenetic tree presented here, the S73 strain was present in a branch separated from the Asian ST283 isolates, which corroborates the findings of Leal et al. (2019). In the study of Leal et al. (2019), serotype III Brazilian isolates were identified as ST-283, using whole-genome MLST approach, and the Brazilian isolates grouped together with fish and human isolates from Asia. Although, the strains from Brazil and Asia (fish isolates) exhibited high genetic diversity, with loci variations ranging from 3.84 to 14.26% (Leal et al., 2019).

S73 is the first serotype III isolated from diseased fish in Brazil, in 2016. It is a multidrug-resistant strain isolated from a streptococcosis outbreak in a northeastern Brazilian fish farm by our group (Chideroli et al., 2017). A total of 32 GIs were predicted in the S73 genome, of which seven were pathogenicity islands (PAI), 10 were metabolic islands (MI), six were resistance islands (RI), five were symbiosis islands (SI), and the remaining four GIs shared the characteristics of two or more island types (Supplementary Table 2).

**FIGURE 2:**
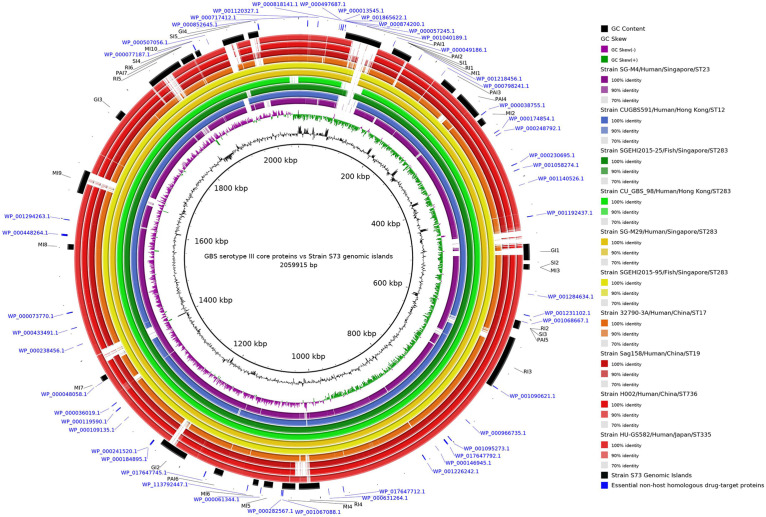
Circular alignment of genomes of representative *Streptococcus agalactiae* serotype III strains. The intensity of the ring color indicates the identity between that genome and the S73 strain, which was used as reference for the alignment. Rings of the same color indicate genomes of strains from the same cluster in the phylogenetic tree. Arranged from the center to the edge: GC content and GC skew of strain S73; strain SG-M4; strain CUGBS591; strain SGEHI2015-25; strain CU_GBS_98; strain SG-M29; strain SGEHI2015-95; strain 32790-3A; strain Sag158; strain H002; strain HU-GS582; predicted genomic islands in strain S73; and essential non-host homologous proteins of strain S73.

The concentric alignment (Figure 2) confirms that the genomes of serotype III isolates, particularly isolates from the same ST or CC, are conserved. It can be noticed that all S73 GIs were present in the genomes of all serotype III isolates, though some of them were not well conserved. Even in strain SGEHI2015-25 (dark green ring), which was isolated from a Singaporean fish in 2015 (Kalimuddin et al., 2017), certain gaps in islands PAI1 and SI5 have been observed. This, along with the discussed possible introduction of serotype III ST283 in South America through trading of live fish from Asia (Barkham et al., 2019; Leal et al., 2019), indicates that the Brazilian strain could have acquired certain genes after the introduction of the strain in the country.

The Orthofinder software identified 1,473 core, 131 shared, and 127 singleton genes. In a study comparing the serotype III isolates only from human hosts, 1,610 core genes were predicted in the pan-genome (Lannes-Costa et al., 2020). The closeness in the sizes of the core genomes observed in the aforementioned study and the present study reinforces the possibility of a strong genomic similarity among serotype III isolates.

### Selection of Drug Targets Based on Subtractive Criteria

To ensure that the probability of drug side-effects is small, it is important that the proteins selected as drug targets do not bear homology to the host’s proteome (Sakharkar et al., 2008). After running a BLASTp search of the core genes against human and *Oreochromis* spp. proteomes, 857 and 773 non-host homologous genes were obtained, respectively. An additional Orthofinder round identified 666 core genes non-homologous to both hosts.

SurfG+ analysis indicated that, among the core non-host homologous proteins, 408 were cytoplasmatic, 154 were membrane proteins, 80 were potentially surface-exposed proteins, and 24 were secreted proteins. Among these, cytoplasmatic proteins were selected as drug targets owing to their important role in cell maintenance (Vilela Rodrigues et al., 2019).

After MHOLline 3D-structure prediction, only 76 proteins were listed as good-quality proteins or above (identity ≥ 50%; *e*-value ≤ 0.3). As essential genes are considered to be great drug targets (Duffield et al., 2010; Mondal et al., 2015), the essentiality of the proteins was assessed by BLASTp against the DEG database; and 48 proteins were found to be essential to the survival of *S. agalactiae* under the established criteria.

Genomic islands are one of the sources of DNA acquired through horizontal gene transfer, and they carry gene clusters that enable the cell to perform certain special activities (Soares et al., 2012). They can contain virulence (pathogenicity), symbiotic, resistance, and metabolic genes (Soares et al., 2016). Owing to their importance in increasing the fitness and pathogenicity of not only the pathogen in which they occur but also a population of organisms, only the proteins encoded by the genes present in the GIs of S73 strain were selected. Although GIs are generally considered a part of the accessory genome, the proteins encoded by the genes present in the GIs have already been filtered as core and essential, indicating that they are probably well-fixated in the genome of this species. Considering that GIs are comprised of mobile genetic elements (in the distant evolutionary past) and that bioinformatic tools can incur minor errors, proteins at the edge of GIs were also selected. Of the 48 essential proteins, three were present in the GIs and the other three were present at the edge of GIs.

Druggable proteins are defined as proteins that bind and are responsive to drug-like molecules (Keller et al., 2006). From a known dataset of druggable features, including size, compactness, and physicochemical properties, of the pockets (Volkamer et al., 2012), the DoGSiteScorer algorithm returned the druggable pockets within the selected proteins with their area, volume, active amino acid residues, and a druggability score. Five of the six selected proteins had pockets with druggability score >0.8 and were selected for molecular docking and drug discovery (Supplementary Table 3). Therefore, the final drug target candidates were proteins WP_000077187, WP_001068667, WP_001090621, WP_001067088, and WP_000282567. The subtractive steps, the number of proteins identified at each step, and the inclusion criteria are summarized in Table 1.

**TABLE 1:** Subtractive genomics steps to obtain potential drug target proteins within the core genome of 24 *Streptococcus agalactiae* serotype III genomes isolated from fish (*n* = 5) and humans (*n* = 19). The inclusion criterium of each step is underlined.

**Steps (software or database)**	**Number of genes/proteins**
**Step 1: Strains’ core genome (Orthofinder)**	
Core genes	1,473
Shared genes	131
Singletons	127
**Step 2.1: Strains’ core gene homology with hosts proteome (BLASTp)**	
Core genes non-homologous to *Homo sapiens* proteome	857
Core genes non-homologous to *Oreochromis* spp. proteome	773
**Step 2.2: Core genes non-homologous to both host genomes (Orthofinder)**	
Core genes	666
Shared genes	176
Singletons	0
**Step 3: Core protein subcellular localization prediction (SurfG+)**	
Cytoplasmic (drug targets)	408
Membrane	154
Potentially surface exposed	80
Secreted	24
**Step 4: Protein structure prediction (Mholline)**	
G0: Non-aligned sequences	69
G1: *e*-value > 10e−5 or ID < 15%	0
G2: *e*-value ≤ 10e−5 and ID ≥ 25%	328
Very high quality sequences: ID ≥ 75%	19
High quality sequences: ≥50% ID < 75%	52
Good quality sequences: ID > 50%	5
Medium to good quality sequences: ≥35% ID < 50%	88
Medium to low quality sequences: ≥25% ID < 35%	93
Low quality sequences: ID ≥ 25%	25
Very low quality sequences: ID ≥ 25%	46
G3: *e*-value ≤ 10e−5 and 15% ≥ ID < 25%	11
**Step 5: Protein essentiality (Database of Essential Genes)**	
Bitscore ≥ 100 and *e*-value ≤ 10e−4	48
**Step 6: Presence within genomic island (GIPSy, BRIG)**	
Proteins encoded by genes present within genomic islands	3
Proteins encoded by genes present at the edge of genomic islands	3
Proteins encoded by genes present outside genomic islands	42
**Step 7: Binding site/pocket detection (DogSiteScorer/ProteinPlus)**	
Proteins having pockets with druggability score ≥ 0.8	5
Proteins having pockets with druggability score < 0.8	1

The protein WP_000077187 showed 100% identity to phosphopentomutase (UniProt accession number Q8CMH7), encoded by *deoB1* gene, of *S. agalactiae* serotype III strain NEM316. This protein was predicted to be encoded by the Metabolic-Island-10 of the S73 strain. Phosphomutases are enzymes responsible for rearranging of phosphate within a substrate molecule. In bacteria, phosphopentomutases catalyze the transfer of phosphate group between C1 and C5 of a pentose, and can act on both ribose- and deoxyribose-phosphates (Tozzi et al., 2006; Panosian et al., 2011); thus, they are directly involved in nucleic acid biosynthesis and energy production in the absence of glucose. Phosphopentomutases can be considered good drug targets because they are distantly related to the human isoform of the enzyme (Panosian et al., 2011). Moreover, a *deoB*-disrupted *Francisella tularensis* mutant has been reported to be less lethal to chicken embryos and defective in entering human phagocytic cells and cultured embryonic kidney cells (Horzempa et al., 2008). There is no record of phosphopentomutase as a drug target in the Drugbank database. These data indicate that phosphopentomutase is a promising target for new drugs as its inhibition can affect both metabolism and virulence of *S. agalactiae*.

The protein WP_001068667 showed 100% identity to the 50S ribosomal protein L19 (Uniprot accession number Q8E6H6), encoded by *rplS* gene, of *S. agalactiae* strain NEM316. According to GIPSy prediction, the gene encoding protein L19 is present at the edge of a GI predicted to be a resistance-, symbiosis-, and pathogenicity-island (-2, -3, and -5, respectively). In *E. coli*, L19 is one of the proteins responsible for joining of the small and large ribosomal subunits and is essential for protein translation and subsequently for cell viability (Persson et al., 1995; Soung et al., 2009). L19 has phosphorylation sites at Ser, Thr, and Tyr amino acids. The phosphorylation of ribosomal proteins can reduce up to 50% of their activity (Mikulík et al., 2001), although it seems that phosphorylated L19 is the active form of the protein that contribute to the assembly and decoding processes (Soung et al., 2009). Moreover, a mutagenic study of the *rplS* gene in *E. coli* revealed that, despite the restraint in translation, the defective mutants did not exhibit problems in 30S–50S association or cell growth (VanNice et al., 2016). There is no record of L19 protein as a drug target in the DrugBank database. Owing to its importance in ribosomal structure, the precise effects of targeting L19 on *S. agalactiae* ribosome structure and function should be addressed in further studies.

The protein WP_001090621 showed 100% identity to RegM protein (Uniprot accession number Q8E0M3), encoded by *sag0707* gene, of *S. agalactiae* serotype V, and 93.1% identity to catabolite control protein A (CcpA; Uniprot accession number Q9A118), encoded by *ccpA* gene, of *Streptococcus pyogenes* serotype M1. In the S73 genome, this protein is localized in Resistance-Island-3. CcpA is a regulatory protein conserved in Gram-positive organisms, and it plays an important role in carbon catabolite repression (a system of rapid adaptation to a preferred carbon source) and expression of virulence factors (Lang et al., 2014; Liao et al., 2017). Giammarinaro and Paton (2002) first described RegM protein while searching for CcpA homologs in *Streptococcus pneumoniae*. In their study, they found that, in addition to the involvement of RegM in carbohydrate catabolism, RegM could regulate the expression of the capsular gene, and *regM* knockout-mutants were less virulent in mice (Giammarinaro and Paton, 2002). In the presence of high glucose concentration, CcpA, encoded by *sag0707*, is downregulated in *S. agalactiae* serotype V (Di Palo et al., 2013). Moreover, targeting CcpA for antimicrobial purposes has been proven to be efficient. Liao et al. (2017) demonstrated that silver ions could bind CcpA and subsequently inhibit the growth of *S. aureus*, toxin expression, and biofilm formation; Huang et al. (2020) reported that a small compound, bis(4-hydroxy-3-methylphenyl) sulfide, inhibited the expression of *ccpA* and α-hemolysin in *S. aureus*. In the DrugBank database, four drugs have been registered to bind to regulators similar to RegM/CcpA in other bacterial species (DrugBank Accession Numbers DB02283, DB01862, DB08297, and DB02430). Virulence regulators remain unexplored in the field of drug discovery, and the available data show that they can serve as great targets (Huang et al., 2020), reinforcing the potential of RegM/CcpA as a drug target in *S. agalactiae*.

The protein WP_001067088 showed 98.7% identity to the flavin mononucleotide (FMN)-binding oxidoreductase (UniProt accession number Q8DZN9), encoded by gene *sag1061*; and protein WP_000282567 showed 97.4% identity to a flavoprotein-related protein (Uniprot accession code Q8DZN7), encoded by *sag1063*, of *S. agalactiae* serotype V strain 2603V/R. Both the genes are predicted to be present in the Metabolic-Island-4 of the S73 strain. FMN and flavin adenine dinucleotide (FAD) function as cofactors of flavoproteins, and flavoproteins are related to several essential and vital functions in living beings (Sebastián et al., 2018). Most prokaryotic FAD synthetases, which synthesize FMN, FAD, and flavoproteins, are different from mammalian FAD synthetases; this allows specific targeting of the prokaryotic proteins and cofactors for antimicrobial purposes (Cremades et al., 2005; Rodríguez-Cárdenas et al., 2016; Sebastián et al., 2018). In *S. agalactiae* serotype III, the flavoprotein type 2 NADH dehydrogenase (NDH-2), described as the only entry point for electrons in the respiratory chain, has already been suggested to be a great drug target (Lencina et al., 2018). As WP_001067088 and WP_000282567 are related to flavoproteins and their cofactors, they can serve as drug targets in *S. agalactiae*. WP_001067088 matched with four drug targets registered in the DrugBank, and 11 different drugs are listed to bind to these drug targets (DrugBank Accession Numbers DB03147, DB03247, DB03461, DB03698, DB01676, DB02060, DB03651, DB04528, DB07373, DB02508, and DB11090). On the other hand, WP_000282567 matched with one drug target in the DrugBank database that can bind to two drugs (DrugBank Accession Numbers DB02431 and DB03403). Information regarding the identified drug targets is summarized in Table 2.

**TABLE 2:** Details of *Streptococcus agalactiae* serotype III core proteins with potential to serve as new drug targets.

**Protein**	**WP_000077187**	**WP_001068667**	**WP_001090621**	**WP_001067088**	**WP_000282567**
**Mholline structure prediction quality**	High	High	High	High	High
**Database of Essential Genes best hit**					
*e*-value	9.78e−160	3.17e−79	0.0	6.59e−30	3.22e−95
Bit score	454	227	672	120	276
**Protein position in strain S73 genome**	Comp (1908924.1910135)	604893.605240	704606.705610	Comp (1051267.1052466)	1053435.1054136
**Strain S73 genomic island (position)**	MI10	RI2/SI3/PAI5	RI3	MI4	MI4
**DogSiteScorer binding site prediction**					
Pocket volume (Å^3^)	577.92	404.54	2349.7	2320.06	456.38
Pocket surface (Å^2^)	810.76	827.89	2977.87	25553.96	649.69
Druggability score	0.82	0.82	0.81	0.81	0.84
**Uniprot database best hit**					
Protein name	Phosphopentomutase	50S ribosomal protein L19	Transcriptional regulator (RegM)	Oxidoreductase, FMN-binding	Flavoprotein-related protein
Organism	*S. agalactiae* ser. III	*S. agalactiae* ser. III	*S. agalactiae* ser. V	*S. agalactiae* ser. V	*S. agalactiae* ser. V
ID	100%	100%	100%	98.7%	97.4%
Uniprot code	Q8CMH7	Q8E6H6	Q8E0M3	Q8DZN9	Q8DZN7
Possible function	Phosphotransfer between C1 and C5 of a pentose	Joining of small and large ribosomal subunits	Related to carbon metabolism and virulence expression	Flavoprotein cofactor	Flavoprotein-related

Although this study focused on targeting serotype III isolates, drug targets homologous to all or the majority of *S. agalactiae* serotypes would be more desirable and economically attractive. A BLASTp search against representative strains from other *S. agalactiae* serotypes showed that these five proteins identified herein are highly conserved in serotypes Ia, Ib, II, IV, V, and VI (Supplementary Table 4). This information indicates that these proteins may serve as drug targets in multiple serotypes.

### Molecular Docking and Compound Identification

Natural compounds have been and still are the main sources of most classes of antibiotics (Genilloud, 2019). Three out of five new compound classes released as antibiotics between 2000 and 2015 for humans were based on natural products (Harvey et al., 2015). Natural compounds have greater chemical diversity as compared to synthetic compounds, and are easily absorbed and metabolized in the body despite their complex structures (Strohl, 2000). Therefore, a library of 5,008 drug-like natural compounds was downloaded from the ZINC database to screen for new candidates against the five proteins identified in *S. agalactiae* serotype III isolates obtained from humans and fish hosts.

**FIGURE 3:**
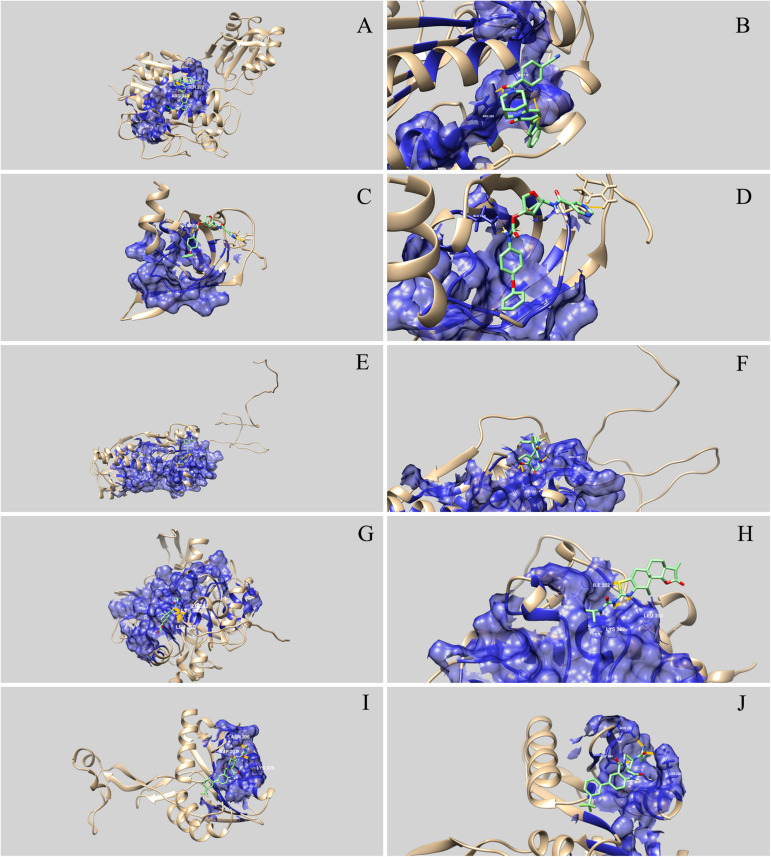
Three-dimensional representation of the interaction between drug-like natural compounds and *Streptococcus agalactiae* serotype III drug target proteins. The blue areas indicate the druggable pocket of the protein. Hydrogen bonds are represented in dark yellow and the amino acid residues involved are identified. **(A)** and **(B)** represent interaction between WP_000077187 (phosphopentomutase) and ZINC05410520; **(C)** and **(D)** represent interaction between WP_001068667 (ribosomal protein L19) and ZINC03838587; **(E)** and **(F)** represent interaction between WP_001090621 (RegM/CcpA) and ZINC04236030; **(G)** and **(H)** represent interaction between WP_001067088 (FMN-binding oxidoreductase) and ZINC03839958; and **(I)** and **(J)** represent interaction between WP_000282567 (flavoprotein-related protein) and ZINC04222225.

Guided by a grid containing the amino acids of the most druggable pocket of each protein (identified by the DoGSiteScorer algorithm), the AutoDock Vina software was employed to screen the ligand library against the five selected drug target proteins. Next, 10 ligands having the lowest binding affinity for each protein were identified using a python script (Supplementary Box 1). These 50 compounds were docked against their respective targets using Chimera software. In this step, the best drug-like molecule to each protein was elected based on the lowest binding affinity and the greatest number of hydrogen bonds between the ligand and the protein (Table 3). The 3D images showing the docked ligands and their respective targets are presented in Figure 3.

**TABLE 3:** Characteristics of the bond between *S. agalactiae* serotype III drug target proteins and drug-like natural compounds. Amino acid residues present in the protein pocket are underlined.

**Protein**		**Compound**		**N° H-bonds**	**Binding affinity (kcal mol^–1^)**	**Amino Acid Residues interacting**
**Annotation in S73 strain genome**	**Uniprot best match (gene)**	**ZINC ID**	**IUPAC name**			
WP_000077187	Phosphopentomutase (*deoB1*)	ZINC05410520	4-[(1R,9S)-5-(1-benzothiophen-2-yl)-6-oxo-7,11-diazatricyclo[7.3.1.02,7] trideca-2,4-diene-11-carbonyl]benzonitrile	3	−6	SER270, ARG320, ARG320
WP_001068667	50S ribosomal protein L19 (*rplS*)	ZINC03838587	[(3S,3aR,6R,6aS)-3-(pyrimidine-5-carbonylamino)-2,3,3a,5,6,6a-hexahydrofuro[3,2-b]furan-6-yl] *N*-(4-phenoxyphenyl) carbamate	4	−5.2	ARG21, ARG21, GLU57, GLU57
WP_001090621	RegM trancriptional regulator (*sag0707*)/ Catabolite control protein A (*ccpA*)	ZINC04236030	(4aS)-3-(furan-2-carbonyl)-9-[3-(trifluoromethyl)phenyl]-2,4,4a,6-tetrahydro-1H-pyrazino[2,1-c][1,4]benzodiazepine-5,11-dione	3	−8.4	ARG136, ARG136, ARG143
WP_001067088	Oxidoreductase, FMN-binding (*sag1061*)	ZINC03839958	1-[(3S,3aS,5aS, 10S,10aS,10bS)-3,5a,10-trimethyl-2-oxo-3,3a,4,5,6,10,10a,10b-octahydro-[1]benzofuro[7,6-f][1,3]benzothiazol-8-yl]-3-propan-2-ylurea	3	−6.4	LEU350, ILE352, LYS382
WP_000282567	Flavoprotein-related protein (*sag1063*)	ZINC04222225	*N*-[(6aS,8S)-6,12-dioxo-2-[3-(trifluoromethyl)phenyl]-5,6a,7,8,9,10-hexahydropyrido[2,1-c][1,4]benzodiazepin-8-yl]pyrazine-2-carboxamide	3	−7.9	ASP203, ASN206, LYS226

Molecular docking analysis revealed that the five selected targets were effective in binding to natural compounds. The best docked protein and ligand, identified based on binding energy and hydrogen bonds, were RegM/CcpA (WP_001090621) and ZINC04236030, respectively. The ligand bound RegM/CcpA with three hydrogen bonds to the amino acids ARG 136 and ARG143, with −8.4 kcal mol^–1^ binding affinity. The involvement of RegM/CcpA in carbohydrate metabolism and regulation of virulence factor expression (Giammarinaro and Paton, 2002; Lang et al., 2014) grounds the potential of targeting this particular protein in further *in vitro* and *in vivo* trials using the ligand herein identified. Promising results have been observed in targeting *S. aureus* Ccpa for antibiotic purposes (Liao et al., 2017; Huang et al., 2020). In addition, RegM/CcpA, being a conserved protein in Gram-positive bacteria (Liao et al., 2017), it can be targeted as broad-range for infections caused by this group of bacteria.

The integration of genome-driven platforms and culture-based approaches may be the key to discover new innovative antibiotics (Genilloud, 2019). With the support of genomic analysis and bioinformatics tools, this study predicted the core conserved proteins in the potentially zoonotic *S. agalactiae* serotype III isolates obtained from human and fish hosts. Following the subtractive criteria, five potential drug targets and drug-like molecules that can bind to them were proposed. Amongst the identified targets, the most promising target, according to the criteria established herein, is the protein WP_001090621, an analog of RegM/CcpA that is involved in both metabolism and virulence regulation; and the best ligand for this target was the compound ZINC04236030. The targets and drugs predicted here can be readily tested *in vitro* and *in vivo*, and they support the development of new strategies for control and treatment of *S. agalactiae* infection that causes serious losses in fish farming and poses a serious threat to public health.

## Data Availability Statement

The datasets presented in this study can be found in online repositories. The names of the repository/repositories and accession number(s) can be found in the article/ Supplementary Material.

## Author Contributions

LF and UP conceived the study. LF, ST, UP, NL-B, and VA performed the research. LF, RC, NF, and ST performed bioinformatics analysis under the supervision of UP and VA. LF wrote the manuscript and produced the figures under the supervision of UP. All authors contributed to discussing the manuscript. All authors have read and approved the final manuscript.

## Conflict of Interest

The authors declare that the research was conducted in the absence of any commercial or financial relationships that could be construed as a potential conflict of interest.

## Footnotes

1 http://www.mholline2.lncc.br/

2 http://www.essentialgene.org/

3 https://proteinsplus.zbh.uni-hamburg.de

4 http://autodock.scripps.edu/
