# Supplementary material for: In silico Prediction of New Drug Candidates Against the Multidrug-Resistant and Potentially Zoonotic Fish Pathogen Serotype III Streptococcus agalactiae
Source: Front Genet. 2020 Aug 28;11:1024. doi: 10.3389/fgene.2020.01024 (PMC7484375; doi:10.3389/fgene.2020.01024)
Supplement: Supplementary file 1 [file Data_Sheet_1.docx]

Supplementary Material

**Supplementary Table 1 Information on the genomes used in this work**

| **SPECIE** | **STRAIN** | **ACCESSION NUMBER** | **SERO**  **TYPE** | **ST** | **HOST** | **ISOLATION SOURCE** | **ORIGIN** | **SIZE (bp)** | **GENE NUMBER** | **PROTEIN NUMBER** | **SEQUENCING TECHNOLOGY** |
| --- | --- | --- | --- | --- | --- | --- | --- | --- | --- | --- | --- |
| *S. agalactiae* | SGEHI2015-25 | NZ_CP025029.1 | III | 283 | fish | muscle (fresh fish) | Singapore | 2054713 | 2173 | 2005 | PacBio |
| *S. agalactiae* | SGEHI2015-113 | NZ_CP025026.1 | III | 283 | fish | muscle (fresh fish) | Singapore | 2043851 | 2156 | 1975 | PacBio |
| *S. agalactiae* | SGEHI2015-107 | NZ_CP025027.1 | III | 283 | fish | muscle (fresh fish) | Singapore | 2016805 | 2119 | 1948 | PacBio |
| *S. agalactiae* | SGEHI2015-95 | NZ_CP025028.1 | III | 283 | fish | muscle (fresh fish) | Singapore | 2116769 | 2248 | 2050 | PacBio |
| *S. agalactiae* | S73 | NZ_CP030845.1 | III | na | fish | Brain | Brazil | 2059915 | 2089 | 1953 | Illumina |
| *S. agalactiae* | 32790-3A | NZ_CP029561.1 | III | 17 | human | blood | China | 2148904 | 2249 | 2022 | PacBio |
| *S. agalactiae* | 874391 | NZ_CP022537.1 | III | 17 | human | vagina | Japan | 2153937 | 2224 | 2034 | PacBio |
| *S. agalactiae* | CU_GBS_08 | NZ_CP010874.1 | III | 283 | human | blood | Hong Kong | 2084511 | 2132 | 1989 | Illumina MiSeq |
| *S. agalactiae* | CU_GBS_98 | NZ_CP010875.1 | III | 283 | human | cerebrospinal fluid | Hong Kong | 2029669 | 2043 | 1908 | Illumina MiSeq |
| *S. agalactiae* | CUGBS591 | NZ_CP021862.1 | III | 12 | human | joint aspirate | Hong Kong | 2227680 | 2362 | 2161 | PacBio |
| *S. agalactiae* | H002 | NZ_CP011329.1 | III | 736 | human | vagina | China | 2147416 | 2143 | 1975 | Illumina Hiseq |
| *S. agalactiae* | HU-GS5823 | NZ_AP018935.1 | III | 335 | human | blood | Japan | 2231314 | 2286 | 2109 | MinION; Illumina Miseq |
| *S. agalactiae* | NEM316 | NC_004368.1 | III | 23 | human | blood | Europa | 2211485 | 2217 | 2083 | na |
| *S. agalactiae* | NGBS128 | NZ_CP012480.1 | III | 17 | human | blood | Canada | 2074179 | 2069 | 1863 | PacBio; Illumina |
| *S. agalactiae* | Sag158 | NZ_CP019979.1 | III | 19 | human | blood | China | 2096882 | 2186 | 2000 | PacBio |
| *S. agalactiae* | Sag27 | NZ_CP031556.1 | III | 19 | human | perianal | China | 2205229 | 2256 | 2075 | PacBio |
| *S. agalactiae* | SG-M1 | NZ_CP012419.2 | III | 283 | human | meningitis | Singapore | 2116811 | 2161 | 2009 | PacBio |
| *S. agalactiae* | SG-M158 | NZ_CP021864.1 | III | 283 | human | blood | Singapore | 2116811 | 2248 | 2075 | PacBio |
| *S. agalactiae* | SG-M163 | NZ_CP021863.1 | III | 283 | human | blood | Singapore | 2116810 | 2246 | 2072 | PacBio |
| *S. agalactiae* | SG-M25 | NZ_CP021867.1 | III | 19 | human | blood | Singapore | 2208337 | 2331 | 2146 | PacBio |
| *S. agalactiae* | SG-M29 | NZ_CP021866.1 | III | 283 | human | blood | Singapore | 2116773 | 2247 | 2072 | PacBio |
| *S. agalactiae* | SG-M4 | NZ_CP021870.1 | III | 23 | human | blood | Singapore | 2072007 | 2153 | 1991 | PacBio |
| *S. agalactiae* | SG-M50 | NZ_CP021865.1 | III | 283 | human | blood | Singapore | 2116810 | 2248 | 2074 | PacBio |
| *S. agalactiae* | SG-M6 | NZ_CP021869.1 | III | 17 | human | blood | Singapore | 2106018 | 2216 | 2013 | PacBio |
| *S. thermophilus* | JIM8232 | NC_017581.1 | na | na | na | Milk | France | 1929905 | 2029 | 1727 | Sanger, SOLiD |

**Supplementary Table 2 Strain S73 genomic islands position**

| **Island ID** | **Start** | **Stop** |
| --- | --- | --- |
| PAI1 | 68244 | 120420 |
| PAI2 | 139689 | 167554 |
| PAI3 | 224027 | 245806 |
| PAI4 | 255259 | 294247 |
| PAI5 | 605240 | 617168 |
| PAI6 | 1140604 | 1153487 |
| PAI7 | 1884594 | 1903430 |
| MI1 | 139689 | 167554 |
| MI2 | 300326 | 310257 |
| MI3 | 523178 | 530912 |
| MI4 | 1034591 | 1053450 |
| MI5 | 1066892 | 1079788 |
| MI6 | 1087956 | 1095663 |
| MI7 | 1360368 | 1367874 |
| MI8 | 1557569 | 1565371 |
| MI9 | 1639836 | 1673580 |
| MI10 | 1907089 | 1914209 |
| RI1 | 139689 | 167554 |
| RI2 | 605240 | 617168 |
| RI3 | 630788 | 707253 |
| RI4 | 999679 | 1029365 |
| RI5 | 1828986 | 1880468 |
| RI6 | 1884594 | 1903430 |
| SI1 | 139689 | 167554 |
| SI2 | 523178 | 530912 |
| SI3 | 605240 | 617168 |
| SI4 | 1884594 | 1903430 |
| SI5 | 1949253 | 1980497 |
| GI1 (PAI, MI, RI e SI) | 494057 | 517583 |
| GI2 (PAI, MI, RI e SI) | 1199007 | 1239807 |
| GI3 (RI e SI) | 1760247 | 1773004 |
| GI4 (PAI, MI, RI e SI) | 1987317 | 2001811 |

PAI: pathogenicity island; MI: metabolic island; RI: resistance island; SI: symbiotic island; GI: genomic island.

**Supplementary Table 3 Information on the druggable pockets identified in *S. agalactiae* serotype III selected drug target proteins**

| **Protein ID** | **Pocket ID** | **Volume (Å³)** | **Surface (Å²)** | **Druggability score** | **Amino acid residues** |
| --- | --- | --- | --- | --- | --- |
| WP_000077187 | P_0 | 577,92 | 810,76 | 0,82 | VAL10, LEU12, ASN52, MET53, LYS55, ILE56, SER77, VAL247, GLY248, LYS249, ASN266, LYS267, SER268, ASN269, SER270, GLY272, ILE273, LEU276, THR292, ASN293, LEU294, VAL295, ASP296, PHE297, CYS313, GLU316, PHE317, ASP318, ARG320, LEU321, GLU323, ILE324, ILE325, MET328, LEU334, ILE336, LEU359, TYR361 |
| WP_001090621 | P_0 | 2349,7 | 2977,87 | 0,81 | GLY68, VAL69, VAL70, ILE71, PRO72, ASN73, ALA75, ASN76, SER77, TYR78, PHE79, LEU82, VAL98, LEU99, ALA100, SER101, SER102, ASP103, GLU104, ASP105, ASP106, LYS108, GLU109, ASN111, VAL112, VAL113, ASN114, THR115, LEU116, PHE117, LYS119, GLN120, VAL121, ASP122, ILE124, PHE126, MET127, GLY128, HIS129, HIS130, LEU131, THA132, GLU133, ILE135, ARG136, GLU138, PHE139, SER140, ARG141, SER142, ARG143, THR144, PRO145, ILE146, VAL147, LEU148, ALA149, GLY150, THR151, VAL152, ASP153, LEU154, LEU158, PRO159, ASN162, ILE163, ASP164, TYR165, ALA168, ALA169, VAL172, ILE173,VAL186, SE187, GLY188, PRO189, LEU190, ILE191, ASP192, ASP193, ASN195, GLY196, ARG199, LEU200, TYR203, VAL219, PHE220, GLU221, TYR224, ARG225, TYR245, VAL246, ALA247, GLU248, ASP249, GLU250, SER275, ASN276, SER278, ILE280, ILE291, GLN293, LEU298, ILE309 |
| WP_001067088 | P_0 | 2320,06 | 2553,96 | 0,81 | LEU26, SER27, PRO28, MET29, VAL30, THR31, ASN32, SER33, SER34, VAL40, ASP44, ILE45, TYR47, ALA48, ARG51, GLN58, ILE59, THR60, GLY61, ALA62, TYR64, GLY69, GLN70, LEU71, PHE72, TYR74, GLY75, PHE76, GLY86, LEU87, LYS89, LEU90, ALA93, LEU103, GLN104, THR106, HIS107, ALA108, PHE165, SER171, ALA173, GLN174, ARG175, LEU176, ARG229, THR231, PRO232, GLU233, GLU234, THR235, ALA264, ILE265, ALA266, AER267, TRP268, GLY169, HIS270, ASP271, VAL272, PHE273, ASN275, THR276, ARG278, LEU287, VAL288, ASN289, MET303, ALA304, THR305, GLY306, GLY307, ILE308, ASN309, LYS313, GLU316, ALA317, HIS320, ALA326, SER327, THR328, PRO329, EU330, VAL331, VAL332, LEU350, ILE352, ILE362, PRO363, LYS364, PHE367, ASP369, ILE370, VAL371, PRO372, LEU373, MET374, ASP375, TYR376, GLY377, GLU378, SER379, LEU380, PRO381, LYS 382 |
| WP_000282567 | P_1 | 456,38 | 649,69 | 0,84 | ILE3, LEU4, ILE5, ILE32, ALA33, TYR36, VAL43, ILE84, LEU85, ILE86, ILE153, LEU154, VAL155, THR182, PHE183, LEU201, LEU202, ASP203, ASN206, VAL207, TYR208, LEU219, ILE220, LYS223, VAL224, LYS226, TYR227 |
| WP_001068667 | P_1 | 404,54 | 827,89 | 0,82 | GLN12, LEU13, ARG14, ASP16, ILE17, PRO18, PHE20, ASP24, VAL26, VAL28, LYS31, VAL47, ILE55, GLU57, TYR59, THR60 VAL61, PHE74, PRO75, ILE76, HIS77, THR78, PRO79, ARG80, VAL81, ASO82, LYS83, ILE84, GLU85 |

**Supplementary Table 4 BLASTp homology of drug target proteins from *Streptococcus agalactiae* serotype III to other serotypes**

| **Strain (Accession number)** | **Serotype** | **Host** | **WP_000077187** | | **WP_001068667** | | **WP_001090621** | | **WP_001067088** | | **WP_000282567** | |
| --- | --- | --- | --- | --- | --- | --- | --- | --- | --- | --- | --- | --- |
|  |  |  | **Coverage (%)** | **ID (%)** | **Coverage (%)** | **ID (%)** | **Coverage (%)** | **ID (%)** | **Coverage (%)** | **ID (%)** | **Coverage (%)** | **ID (%)** |
| *S. agalactiae* A909 (NC_007432) | Ia | Human | 100 | 100 | 100 | 100 | 99 | 99 | 99 | 99 | 99 | 98 |
| *S. agalactiae* 515 (NZ_CP051004) | Ia | Human | 100 | 99.75 | 100 | 100 | 100 | 100 | 99 | 99 | 100 | 98.68 |
| *S. agalactiae* GD201008-001 (NC_018646) | Ia | Fish | 100 | 100 | 100 | 100 | 100 | 99.70 | 100 | 99 | 100 | 98.25 |
| *S. agalactiae* ZQ0910 (NZ_CP049938) | Ia | Fish | 100 | 99.75 | 100 | 100 | 100 | 99.70 | 100 | 99 | 100 | 98.25 |
| *S. agalactiae* H36B (GCA_000167795) | Ib | Human | 100 | 100 | 100 | 100 | 100 | 100 | 99 | 99 | 99 | 98 |
| *S. agalactiae* 138P (CP007482) | Ib | Fish | 75 | 98.62 | 100 | 100 | 100 | 100 | 98 | 98 | 99 | 98 |
| *S. agalactiae* 18RS21 (GCA_000167715) | II | Human | 100 | 100 | 100 | 100 | 100 | 100 | 100 | 100 | 100 | 99 |
| *S. agalactiae* GBS1-NY (NZ_CP007570) | II | Human | 100 | 100 | 100 | 100 | 100 | 96.11 | 100 | 100 | 100 | 99.56 |
| *S. agalactiae* NEM316 (NC_004368) | III | Human | 100 | 100 | 100 | 100 | 100 | 100 | 100 | 100 | 100 | 100 |
| *S. agalactiae* CCUG 19094 (GCA_000288675) | III | Human | 99 | 99 | 100 | 100 | 100 | 100 | 100 | 98.75 | 100 | 97.37 |
| *S. agalactiae* COH1 (NZ_HG939456) | III | Human | 100 | 99.75 | 100 | 100 | 100 | 100 | 100 | 98.75 | 99 | 99 |
| *S. agalactiae* NGBS061 (NZ_CP007631) | IV | Human | 100 | 100 | 100 | 100 | 100 | 96.41 | 100 | 100 | 100 | 99.56 |
| *S. agalactiae* NGBS572 (NZ_CP007632) | IV | Human | 100 | 100 | 100 | 100 | 100 | 96.41 | 100 | 98.75 | 99 | 98.67 |
| *S. agalactiae* 2603V/R (NC_004116) | V | Human | 100 | 100 | 100 | 100 | 100 | 100 | 99 | 99 | 98 | 97 |
| *S. agalactiae* CJB111 (GCA_000167755) | V | Human | 100 | 100 | 100 | 100 | 94 | 100 | 100 | 100 | 100 | 99.56 |
| *S. agalactiae* 09mas018883 (NC_021485) | V | Bovine | 100 | 100 | 100 | 100 | 100 | 100 | 100 | 100 | 100 | 99.56 |
| *S. agalactiae* ILRI112 (HF952106) | VI | Dromedary | 100 | 99 | 100 | 100 | 99 | 99 | 99 | 99 | 98 | 97 |
| *S. agalactiae* GBS-M002 (NZ_CP013908) | VI | Human | 100 | 100 | 100 | 100 | 100 | 96.41 | 100 | 99.25 | 100 | 98.25 |

**Supplementary Figure 1** Similarity heatmap based on whole genomes of *Streptococcus agalactiae* serotype III isolates from human and fish hosts


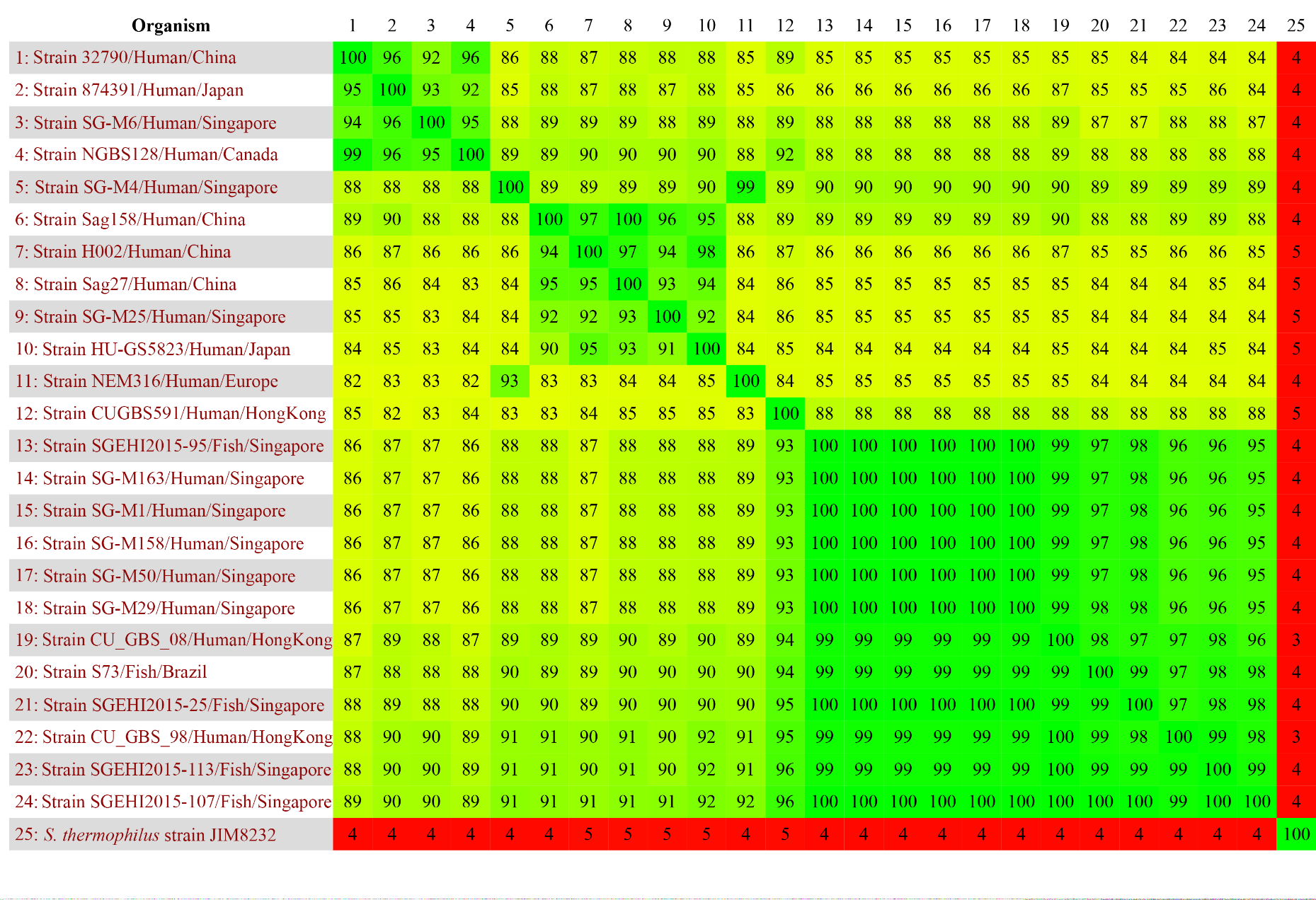


**Supplementary Box 1 Top 10 ligands for each drug-target identified in *S. agalactiae* serotype III. Ligands identified in red were screened as the best docking to its target.**

|  |  |  |  |  |  |  |  |  |
| --- | --- | --- | --- | --- | --- | --- | --- | --- |
|  | **Protein** | **WP_000077187** | |  | **Protein** | **WP_001068667** | |  |
|  | **Best 10 ligands** | ZINC05410520 | ZINC04237087 |  | **Best 10 ligands** | ZINC03838445 | ZINC03838585 |  |
|  |  | ZINC04235928 | ZINC04259075 |  |  | ZINC03838485 | ZINC03838587 |  |
|  |  | ZINC04260398 | ZINC04259499 |  |  | ZINC03838486 | ZINC03838628 |  |
|  |  | ZINC04277699 | ZINC08300419 |  |  | ZINC03838531 | ZINC03838631 |  |
|  |  | ZINC04236005 | ZINC04222225 |  |  | ZINC03838534 | ZINC03838693 |  |
|  |  |  |  |  |  |  |  |  |
|  | **Protein** | **WP_001090621** | |  | **Protein** | **WP_001067088** | |  |
|  | **Best 10 ligands** | ZINC04237100 | ZINC04258935 |  | **Best 10 ligands** | ZINC04237162 | ZINC67897676 |  |
|  |  | ZINC04235882 | ZINC04222703 |  |  | ZINC03839958 | ZINC04237238 |  |
|  |  | ZINC04237087 | ZINC04236304 |  |  | ZINC04259535 | ZINC04259049 |  |
|  |  | ZINC04277699 | ZINC04237085 |  |  | ZINC05396545 | ZINC08296267 |  |
|  |  | ZINC04258917 | ZINC04236030 |  |  | ZINC06137747 | ZINC08300264 |  |
|  |  |  |  |  |  |  |  |  |
|  | **Protein** | **WP_000282567** | |  |  |  |  |  |
|  | **Best 10 ligands** | ZINC08299978 | ZINC04222225 |  |  |  |  |  |
|  |  | ZINC04236081 | ZINC08300249 |  |  |  |  |  |
|  |  | ZINC04222703 | ZINC04235880 |  |  |  |  |  |
|  |  | ZINC04236001 | ZINC04235966 |  |  |  |  |  |
|  |  | ZINC04236036 | ZINC04236020 |  |  |  |  |  |
|  |  |  |  |  |  |  |  |  |
